# Ursolic acid improves growth performance, intestinal health, and antioxidant status in broilers by regulating lipid metabolism and the KEAP1-NRF2 pathway

**DOI:** 10.1093/jas/skaf333

**Published:** 2025-09-29

**Authors:** Man Zhao, Fengyang Wu, Chong Li, Linna Guo, Baojiang Chen, Fengxia Wang, Shudong Liu, Shuaijuan Han

**Affiliations:** College of Animal Science and Technology, Hebei Agricultural University, Baoding, 071001, China; College of Food Science and Technology, Hebei Agricultural University, Baoding, 071001, China; College of Animal Science and Technology, Hebei Agricultural University, Baoding, 071001, China; College of Animal Science and Technology, Hebei Agricultural University, Baoding, 071001, China; College of Animal Science and Technology, Hebei Agricultural University, Baoding, 071001, China; College of Animal Science and Technology, Hebei Agricultural University, Baoding, 071001, China; College of Animal Science and Technology, Hebei Agricultural University, Baoding, 071001, China; College of Animal Science and Technology, Hebei Agricultural University, Baoding, 071001, China

**Keywords:** antibiotic alternative, antioxidant capacity, broiler, growth performance, gut health, ursolic acid

## Abstract

In response to the urgent need for alternatives to antibiotics in poultry production, this study evaluated the effects of ursolic acid (UA), a plant-derived triterpenoid, on growth performance, metabolism, intestinal function, and antioxidant capacity in broilers. A total of 320 Cobb broilers were randomly divided into four groups: a control group and three groups supplemented with UA at 0, 50, 200, or 400 mg/kg of diet, with 8 replicates of 10 birds each, over a 42-day period. Body weight and feed intake were measured on days 21 and 42. On day 42, samples were collected for enzyme-linked immunosorbent assay (ELISA), untargeted metabolomics, hematoxylin–eosin (H&E) staining, meat quality assessment, and quantitative real-time PCR (qRT-PCR) analyses. Data were analyzed using one-way analysis of variance (ANOVA) in SPSS, and orthogonal contrasts were used to assess linear and quadratic responses to UA. The results showed that UA supplementation significantly enhanced growth and slaughter performance (*P *< 0.05), increased serum total protein (*P *< 0.05), and reduced serum triglycerides (*P *< 0.05). Untargeted metabolomics indicated that 200 mg/kg UA modulated lipid metabolism via α-linolenic acid and glycerophospholipid pathways. Additionally, this dose significantly improved the villus height-to-crypt depth ratio (VH/CD) and ileal protease activity (*P *< 0.05). Both 200 and 400 mg/kg UA reduced the b_45min_ value of breast muscle (*P *< 0.05). Furthermore, UA supplementation elevated antioxidant enzyme activities in serum and ileum (*P *< 0.05). At the molecular level, 200 mg/kg UA upregulated expression of amino acid and fatty acid transporter genes, downregulated kelch-like ECH-associated protein 1 (*KEAP1*) (*P *< 0.05), and enhanced expression of catalase (*CAT*), superoxide dismutase 1 (*SOD1*), nuclear factor E2-related factor 2 (*NRF2*), and NAD(P)H: quinone oxidoreductase (*NQO1*) (*P *< 0.05). In conclusion, UA effectively improved growth performance, metabolic health, intestinal function, and antioxidant capacity in broilers, demonstrating its potential as a safe and effective alternative to conventional growth promoters in poultry production.

## Teaser

The manuscript reports that ursolic acid supplementation improves broiler health and productivity through multiple mechanisms, including enhanced nutrient metabolism, gut function, and antioxidant capacity, suggesting its potential as an effective antibiotic replacement.

## Graphical abstract

**Graphical Abstract:**
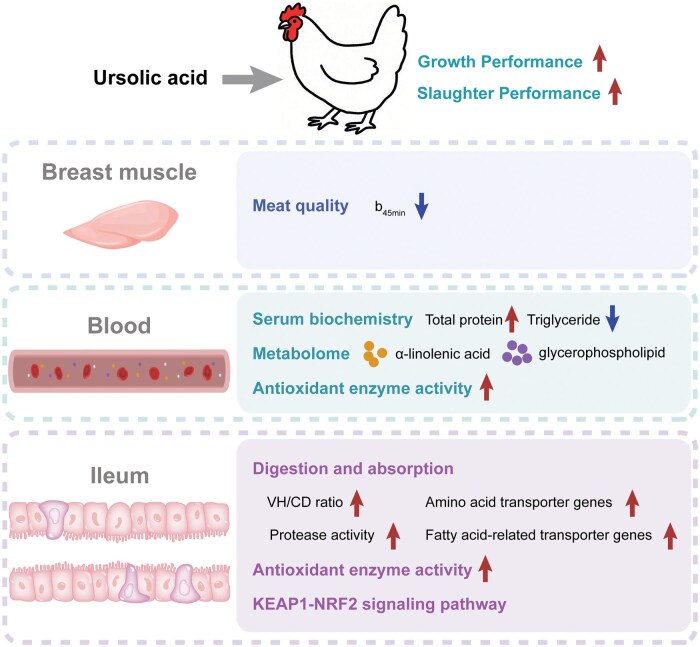

## Introduction

Ursolic acid (UA), a pentacyclic triterpenoid compound, is abundantly found in numerous plant species and food sources (Cargnin and Gnoatto, 2017; López-Hortas et al., 2018). UA has garnered significant scientific interest owing to its multifaceted pharmacological effects, such as its ability to combat cancer, reduce inflammation, and inhibit microbial growth (Cargnin and Gnoatto, 2017).

Modern intensive broiler farming is marked by high feeding densities and rapid growth rates, making it highly vulnerable to various diseases and stressors. These challenges can directly impair production performance and result in significant economic losses (Adewole et al., 2021). Historically, antibiotics have been instrumental in enhancing growth and disease prevention in broilers (Kim et al., 2024). Given the progressive decline and expected cessation of preventive antibiotic administration in poultry production, identifying effective alternatives has become a critical priority to safeguard broiler health and maintain productivity levels (Pu et al., 2024). Among the most promising alternatives are plant extracts, particularly those with bioactive compounds, as they are non-toxic and free from residual side effects while also promoting animal growth (Veiga et al., 2020).

Notably, prior studies have demonstrated that UA can regulate immune balance and enhance the levels of antioxidant enzymes in the body, highlighting its potential as a potent antioxidant (Zhao et al., 2023, 2024). Furthermore, UA has been shown to enhance lipid metabolism in the muscle of fattening pigs by modulating genes related to fatty acid transport (Zhang et al., 2023), and recent studies in chickens indicate its beneficial effects on broiler growth performance (Zhang et al., 2024). Despite these promising findings, there remains a lack of comprehensive research on UA’s application in poultry, and its effects on broiler growth and health are not fully understood. Given the increasing consumer preferences for antibiotic-free agricultural products, a comprehensive investigation of UA’s functional potential and underlying biological mechanisms is critically needed.

Therefore, this study aimed to assess how varying concentrations of UA influence several key aspects in broiler chickens, including growth performance, body metabolism, intestinal digestion and absorption, and antioxidant capacity. Additionally, the study sought to explore the underlying mechanisms of UA’s effects and determine the optimal supplementation level. The results are intended to establish a solid theoretical basis and provide practical insights regarding UA’s role as an innovative feed additive, promoting sustainability within the poultry industry.

## Materials and Methods

### Experimental design and animal management

The experimental protocols were approved by the Animal Care and Use Committee of Hebei Agriculture University (Baoding, China) under Protocol No. 2022003. A total of 320 male Cobb broilers, aged one day, were procured from Jiuxing Farming Co., Ltd (Baoding, China) and randomly allocated into four experimental groups: a control group (CON) and three treatment groups (UA 50, UA 200, and UA 400). Each group comprised eight replicates, each containing ten birds. The CON group was provided with a basal diet, while the treatment groups were administered the same diet enriched with 50, 200, or 400 mg/kg of UA (90% purity, sourced from Chenguang Bio-Technology Co., Ltd., Handan, China). The UA supplementation levels in this experiment were scientifically determined and optimized based on extensive preliminary data and our research group’s prior experimental results (Zhang et al., 2024; Zhao et al., 2024). The trial spanned 42 days with unrestricted feed and water supplies. The basal diet was prepared following the Chinese feeding standards for chickens (NY/T 33-2004) (Supplementary Table S1). The experimental diets did not contain any antibiotics. Experimental management is described previously (Wu et al., 2024).

### Performance records and sample preparation

Feed intake and body weight of each replicate were measured on days 21 and 42. Average body weight (ABW), average daily gain (ADG), average daily feed intake (ADFI), and feed conversion ratio (FCR) were calculated based on data from each trial phase (Peng et al., 2024).

Upon completion of the experiment, one randomly selected individual from each replicate was used for sample collection. Blood was drawn from the wing vein, centrifuged for 10 min (3,000 × g), and the resulting serum was preserved at −80°C. After euthanasia, the abdominal cavity was accessed. Carcasses and organs were weighed. Subsequently, slaughter performance was determined. The detailed methods for determining slaughter performance are shown in Supplementary Material S1. Muscle samples from the breast and leg were stripped and preserved at 4°C for subsequent meat quality determination. From the ileum of each broiler, a 1-cm section was excised and preserved in 4% paraformaldehyde for subsequent morphological examination. Two segments of ileal tissue and one sample of ileal content were collected and stored frozen at −80°C. The ileal tissue samples were subsequently allocated for the determination of antioxidant enzyme activities and quantitative real-time PCR (qRT-PCR) analysis, respectively, while the ileal content was used for measuring digestive enzyme activities.

### Serum biochemical indexes

To evaluate the effects of UA on the overall metabolic health and nutritional status of broilers, serum concentrations of albumin (ALB), glucose (GLU), total protein (TP), globulin (GLB), low-density lipoprotein (LDL), total cholesterol (T-CHO), high-density lipoprotein (HDL), and triglyceride (TG) were determined using an automated biochemistry analyzer (7020, Hitachi High-Technologies Corporation, Tokyo, Japan).

### Untargeted metabolomics analysis

Metabolites were extracted using a methanol-water mixture. Data acquisition was carried out utilizing a SCIEX UPLC-Triple TOF 6600 platform. Initial processing of raw data, including peak detection and alignment, was conducted with Progenesis QI 2.3 software. Metabolites detected in no less than 80% of the samples were retained, and the data were log10-transformed prior. Orthogonal partial least squares discriminant analysis (OPLS-DA) was implemented via the “ropls” package (version 1.6.2). Differential metabolites were identified based on a variable importance projection (VIP) > 1 and false discovery rate (FDR) < 0.05. These metabolites were subsequently annotated using the Human Metabolome Database (HMDB) and the Kyoto Encyclopedia of Genes and Genomes (KEGG) databases. Pathway enrichment analysis was then executed using the KEGG database.

### Intestinal morphology

Histomorphological observation was performed on the fixed and processed ileal tissues. The detailed material is given in Supplementary Material S2.

### Digestive enzyme assays

The activities of amylase, trypsin, and lipase in ileal contents were quantitatively determined using commercial enzyme-linked immunosorbent assay (ELISA) kits (Jiangsu Meimian Industrial Co., Ltd., Yancheng, China). Comprehensive details are provided in Supplementary Material S3.

### Meat quality

pH measurements of breast and leg muscle tissues were taken at 45 min, 24 h, and 48 h post-slaughter using a pH meter (Shenzhen Jige Electromechanical Equipment Co., Ltd, Shenzhen, China), with three measurements per sample. Colorimetric analysis, including redness (a*), yellowness (b*), and brightness (L*), was performed on the same samples at identical time points using a CR410 colorimeter (Konica Minolta Sensing, Japan; Zhang et al., 2017). Additionally, cooking loss, drip loss, and shear force were evaluated in breast and leg muscle tissues 24 h after slaughter, following established protocols (Yang et al., 2024). The detailed material is given in Supplementary Material S4.

### Antioxidant indexes

Frozen ileum tissue samples were processed according to the method described in Supplementary Material S3. Antioxidant indexes, including catalase (CAT), glutathione peroxidase (GSH-Px), superoxide dismutase (SOD), total antioxidant capacity (T-AOC), and malondialdehyde (MDA), were measured in both serum and ileum tissue homogenates using ELISA kits (Nanjing Jiancheng Bioengineering Institute, Nanjing, China).

### qRT-PCR analysis

We comprehensively evaluated the effects of UA on nutrient metabolism and antioxidant status in broilers through a multi-faceted assessment of growth performance, blood biochemistry, intestinal morphology, digestive enzyme activity, and antioxidant enzyme activity. To elucidate the underlying molecular mechanisms by which UA modulates nutrient absorption and antioxidant function in the ileum, we conducted qRT-PCR analysis on ileal tissues following established methods (Zhao et al., 2024). Detailed methodologies are available in Supplementary Material S5, and the corresponding primer sequences are provided in Supplementary Table S3.

### Statistical analysis

Statistical analysis was performed using one-way analysis of variance (ANOVA) in SPSS (version 26). When a significant main effect was observed, post-hoc comparisons were conducted using Tukey's honestly significant difference (HSD) test to control the family-wise error rate across all pairwise comparisons. This test was selected for its suitability with equal sample sizes across groups, as in the present design. Data are presented as means ± SEM, and differences were considered statistically significant at *P *< 0.05. Nonparametric tests were performed on the mortality rate and significance was assessed by the χ^2^ value and set at *P *< 0.05. Additionally, orthogonal polynomial contrasts were used to assess linear and quadratic trends in response to dietary UA levels.

## Results

### Growth performance and slaughter performance

The effects of UA at various concentrations on the growth performance of broilers are detailed in Table 1. Dietary supplementation with 50, 200, and 400 mg/kg UA increased the ABW of broilers by 6.91%, 7.12%, and 7.01%, respectively, at 21 days of age (*P *< 0.05). Furthermore, at 42 days of age, the ABW of broilers receiving 200 mg/kg UA was 7.62% higher than that of the CON group (*P *< 0.05). As the dietary UA level increased, the ABW at 21 days exhibited both linear and quadratic trends, while a significant quadratic trend persisted at 42 days (*P *< 0.05). From day 1 to day 21, UA at 50, 200, and 400 mg/kg increased the ADG by 7.15%, 7.39%, and 7.26%, respectively (*P *< 0.05), with both linear and quadratic dose-response relationships observed (*P *< 0.05). Additionally, during the period of 22 to 42 days, the FCR in the 200 mg/kg UA group decreased by 7.65% (*P *< 0.05), showing a significant quadratic effect with increasing dosage (*P *< 0.05). Over the entire experimental period, 200 mg/kg UA increased ADG by 7.76% (*P *< 0.05) and exhibited both linear and quadratic effects (*P *< 0.05). The FCR was reduced by 5.42% in the 200 mg/kg UA group (*P *< 0.05), with both linear and quadratic effects being significant (*P *< 0.05). Dietary UA supplementation had no significant effect on broiler mortality or organ indices (Supplementary Tables S3 and S4, *P *> 0.05).

**Table 1.** Effects of ursolic acid at graded concentrations on growth performance of broiler chickens (*n *= 8)

Items	Groups				SEM	*P*-values		
	CON	UA 50	UA 200	UA 400		ANOVA	Linear	Quadratic
ABW (g)								
1 d	42.30	42.90	42.65	42.75	0.095	0.891	0.339	0.422
21 d	1000.19^b^	1069.32^a^	1071.39^a^	1070.31^a^	9.331	0.008	0.027	0.047
42 d	2316.25^b^	2432.04^ab^	2492.81^a^	2419.50^ab^	18.492	0.003	0.068	0.002
ADG (g)								
1–21 d	45.62^b^	48.88^a^	48.99^a^	48.93^a^	0.443	0.008	0.028	0.047
22–42 d	62.67	64.89	67.69	64.25	0.756	0.119	0.489	0.022
1–42 d	54.14^b^	56.88^ab^	58.34^a^	56.59^ab^	0.440	0.004	0.068	0.002
ADFI (g)								
1–21 d	65.07	67.44	69.57	66.41	1.011	0.467	0.731	0.128
22–42 d	114.67	114.12	113.90	113.85	0.821	0.986	0.763	0.854
1–42 d	89.87	90.78	91.73	90.13	0.693	0.799	0.950	0.325
FCR								
1–21 d	1.43	1.38	1.42	1.36	0.018	0.528	0.332	0.631
22–42 d	1.83^a^	1.76^ab^	1.69^b^	1.78^ab^	0.019	0.039	0.290	0.007
1–42 d	1.66^a^	1.60^ab^	1.57^b^	1.59^ab^	0.011	0.023	0.056	0.024

Abbreviations: CON, basal diet; UA 50, basal diet + 50 mg/kg ursolic acid; UA 200, basal diet + 200 mg/kg ursolic acid; UA 400, basal diet + 400 mg/kg ursolic acid; SEM, standard error of means; ABW, average body weight; ADG, average daily gain; ADFI, average daily feed intake; FCR, feed conversion ratio.

Values with different superscript letters are statistically different (*P *< 0.05).

Table 2 summarizes the effects of different UA levels on slaughter performance. Dietary supplementation with 200 mg/kg UA increased the half-eviscerated yield and eviscerated yield by 3.89% and 7.56%, respectively, in 42-day-old broilers (*P *< 0.05), with a significant quadratic effect observed as the dosage increased (*P *< 0.05). Moreover, UA at 50, 200, and 400 mg/kg increased the breast muscle yield by 17.10%, 20.63%, and 17.06%, respectively (*P *< 0.05), with significant linear and quadratic effects detected in response to increasing dosage (*P *< 0.05).

**Table 2.** Effects of ursolic acid at graded concentrations on slaughter performance of broiler chickens (*n *= 8).

Items	Groups				SEM	*P*-values		
	CON	UA 50	UA 200	UA 400		ANOVA	Linear	Quadratic
Dressing percentage (%)	91.52	93.68	92.15	91.78	0.421	0.272	0.549	0.502
Half-eviscerated yield (%)	84.58^b^	86.22^ab^	87.87^a^	85.47^ab^	0.429	0.038	0.542	0.005
Eviscerated yield (%)	72.05^b^	73.64^b^	77.50^a^	73.83^b^	0.528	0.001	0.089	<0.001
Breast muscle yield (%)	26.37^b^	30.88^a^	31.81^a^	30.87^a^	0.623	0.004	0.025	0.011
Leg muscle yield (%)	20.00	19.75	19.33	20.48	0.325	0.670	0.575	0.275
Abdominal fat yield (%)	1.36	1.46	1.37	1.66	0.091	0.651	0.307	0.591

Abbreviations: CON, basal diet; UA 50, basal diet + 50 mg/kg ursolic acid; UA 200, basal diet + 200 mg/kg ursolic acid; UA 400, basal diet + 400 mg/kg ursolic acid; SEM, standard error of means.

Values with different superscript letters are statistically different (*P *< 0.05).

### Serum biochemical indices

The effects of different UA concentrations on serum biochemical parameters are presented in Table 3. UA at 200 mg/kg increased serum TP levels by 8.23% (*P *< 0.05), while UA at 50 and 200 mg/kg reduced TG levels by 32.20% and 28.81%, respectively (*P *< 0.05). Additionally, broilers in the 200 mg/kg UA group exhibited a 25.53% reduction in serum LDL levels compared to the CON group (*P *< 0.05). Significant quadratic effects were observed for TP, TG, and LDL in response to increasing UA supplementation (*P *< 0.05). Given the significant positive effects of the 200 mg/kg UA group (hereinafter referred to as the UA group) on growth indicators, slaughter results, and serum biochemical parameters, this group was selected for further serum untargeted metabolomics analysis.

**Table 3.** Effects of ursolic acid at graded concentrations on serum biochemical indices of broiler chickens (*n *= 8).

Items	Groups				SEM	*P*-values		
	CON	UA 50	UA 200	UA 400		ANOVA	Linear	Quadratic
TP (g/L)	26.98^b^	28.38^ab^	29.20^a^	27.35^b^	0.226	0.010	0.355	0.002
ALB (g/L)	16.67	17.23	17.17	17.60	0.312	0.769	0.338	0.916
GLB (g/L)	10.32	11.72	12.03	9.75	0.379	0.134	0.688	0.025
GLU (mmol/L)	16.73	14.76	16.14	15.57	0.324	0.206	0.479	0.294
TG (mmol/L)	0.59^a^	0.40^b^	0.42^b^	0.58^a^	0.011	<0.001	0.973	<0.001
T-CHO (mmol/L)	3.40	3.51	2.97	3.59	0.091	0.062	0.962	0.139
HDL (mmol/L)	2.03	2.04	2.01	2.01	0.018	0.899	0.599	0.915
LDL (mmol/L)	0.94^a^	0.74^ab^	0.70^b^	0.92^ab^	0.034	0.012	0.660	0.001

Abbreviations: CON, basal diet; UA 50, basal diet + 50 mg/kg ursolic acid; UA 200, basal diet + 200 mg/kg ursolic acid; UA 400, basal diet + 400 mg/kg ursolic acid; SEM, standard error of means; TP: total protein; ALB: albumin; GLB: globulin; GLU: glucose; TG: triglyceride; T-CHO: total cholesterol; HDL: high-density lipoprotein; LDL: low-density lipoprotein.

Values with different superscript letters are statistically different (*P *< 0.05).

### Untargeted metabolomics analysis

**Figure 1.**
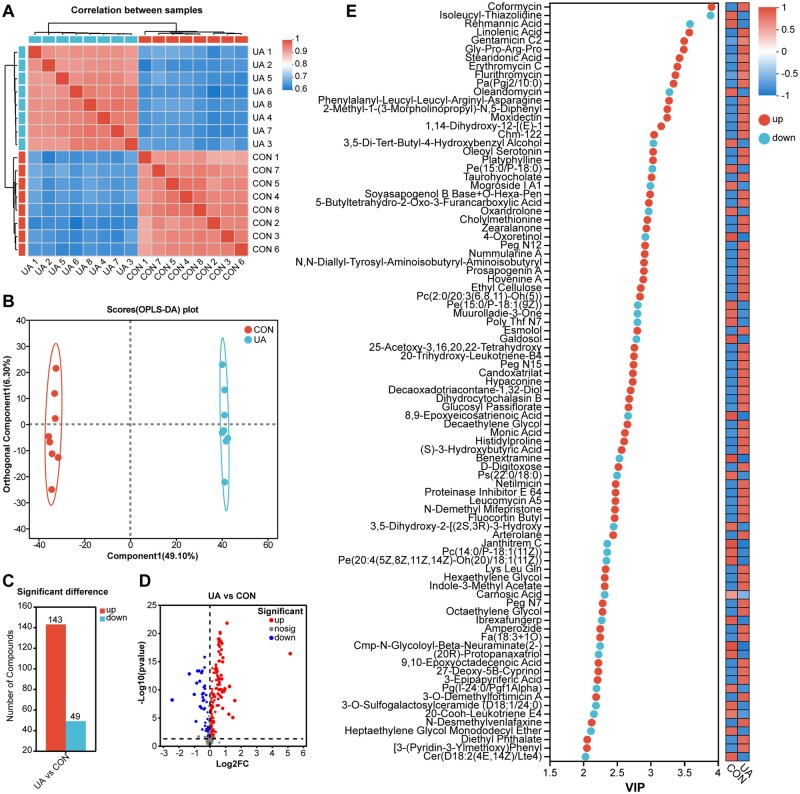
Effects of ursolic acid on serum metabolome of broiler chickens. (A) Heatmap of sample correlation. (B) Orthogonal partial least squares discriminant analysis (OPLS-DA) analysis. (C) Histogram of differential metabolites. (D) Volcano map. (E) Variable importance projection (VIP) analysis of differential metabolites. *n *= 8. CON, basal diet; UA, basal diet + 200 mg/kg ursolic acid.

**Figure 2.**
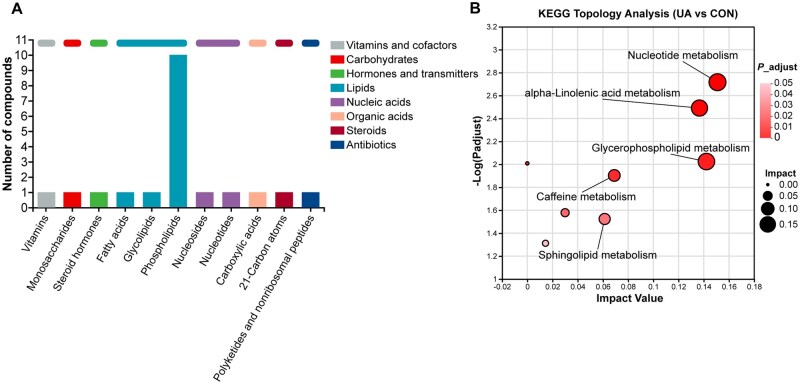
Kyoto Encyclopedia of Genes and Genomes (KEGG) classification of differential metabolites. (A) Differential metabolite KEGG compound classification. (B) Enrichment analysis of KEGG pathway of differential metabolites. *n *= 8. CON, basal diet; UA, basal diet + 200 mg/kg ursolic acid.

**Figure 3.**
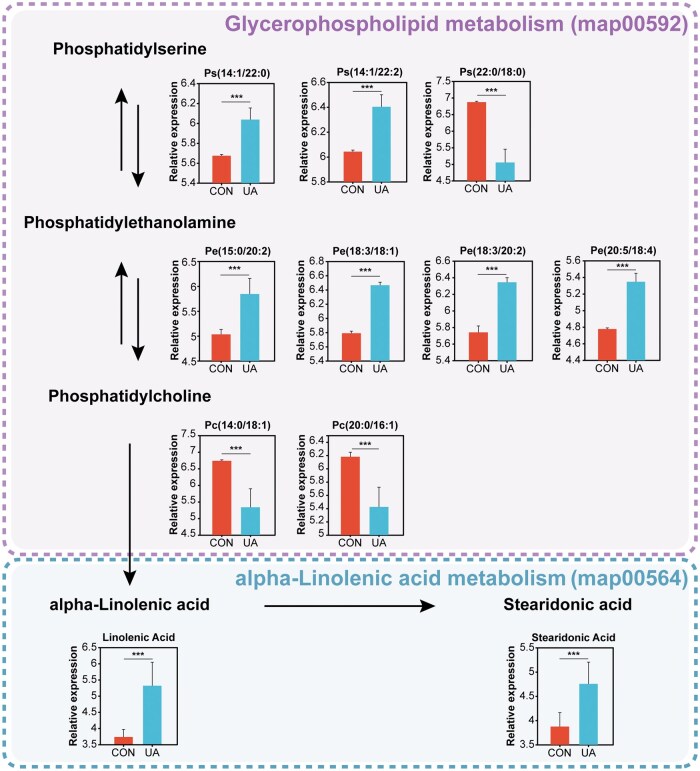
Ursolic acid regulates glycerophospholipid metabolism and α-linolenic acid metabolism in broiler chickens. The pathway was constructed based on KEGG pathway maps, where arrows represent enzyme-catalyzed reactions. *n *= 8. *** FDR < 0.001. CON, basal diet; UA, basal diet + 200 mg/kg ursolic acid.

To examine the impact of dietary UA on serum metabolism in broilers, an untargeted metabolomics approach was applied to analyze serum samples. The results of the sample correlation analysis (Figure 1A) and OPLS-DA (Figure 1B) demonstrated clear differentiation in metabolic patterns between the CON and UA groups. A total of 192 metabolites showed significant differences between the two groups, with 49 metabolites more abundant in the CON group and 143 metabolites more abundant in the UA group (VIP > 1, FDR < 0.05) (Figure 1C and D). Among the top 5 metabolites based on VIP values, isoleucyl-thiazolidine and rehmannic acid were notably higher in the CON group, while coformycin, linolenic acid, and gentamicin C2 were notably elevated in the UA group (Figure 1E). Classification of HMDB compounds revealed that the differential metabolites primarily belonged to organooxygen compounds (22), fatty acyls (17) and carboxylic acids and derivatives (16), and at the class level (Figure S1A). At the subclass level, the differential metabolites were mainly amino acids, peptides, and analogues (15) and carbohydrates and carbohydrate conjugates (15) (Figure S1B). KEGG pathway analysis indicated that lipids (12), particularly phospholipids (10), glycolipids (1), and fatty acids (1), were the most prominent categories (Figure 2A). Differential metabolites were significantly enriched in eight pathways, including nucleotide metabolism, α-linolenic acid metabolism, and glycerophospholipid metabolism (*P *< 0.05) (Figure 2B). Mapping of differential metabolites involved in glycerophospholipid metabolism and α-linolenic acid metabolism revealed their distribution across these two pathways (Figure 3). Compared to the CON group, six metabolites of glycerophospholipid metabolism in the UA group were significantly upregulated, including phosphatidylserine (PS; ps(14:1/22:0), ps(14:1/22:2)) and phosphatidylethanolamine (PE; pe(15:0/20:2), pe(18:3/18:1), pe(18:3/20:2), pe(20:5/18:4)), while three metabolites, including phosphatidylserine (PS; ps(22:0/18:0)) and phosphatidylcholine (PC; Pc(14:0/16:1), pc(20:0/16:1)), were significantly downregulated (FDR < 0.05). Additionally, the two metabolites of α-linolenic acid metabolism, α-linolenic acid and staridonic acid, showed significantly higher levels in the UA group relative to the CON group (FDR < 0.05).

### Ileum morphology and digestive enzyme activity

Table 4 illustrates the effects of different UA concentrations on ileal morphology of broilers. At 42 days of age, supplementation with 200 mg/kg UA significantly increased the VH/CD ratio (*P *< 0.05), from 3.83 to 4.36, with a significant quadratic effect as the dosage increased (*P *< 0.05). Furthermore, trypsin activity in the ileum of broilers in the UA 200 group was 14.33% higher than that of the CON group (*P *< 0.05), showing significant linear and quadratic effects with increasing dosage (*P *< 0.05, Table 5).

**Table 4.** Effects of ursolic acid at graded concentrations on ileal morphology of broiler chickens (*n *= 8).

Items	Groups				SEM	*P*-values		
	CON	UA 50	UA 200	UA 400		ANOVA	Linear	Quadratic
Villus height (µm)	567.22	591.92	609.25	591.55	6.692	0.193	0.168	0.120
Crypt depth (µm)	148.68	143.25	140.30	157.45	2.412	0.072	0.304	0.020
VH/CD	3.83b	4.17ab	4.36a	3.77b	0.069	0.004	0.979	0.001
Villus width (µm)	93.49	97.42	94.43	96.90	2.122	0.908	0.739	0.871
Intestinal wall thickness (µm)	219.67	196.88	137.70	174.58	11.521	0.062	0.077	0.186

Abbreviations: CON, basal diet; UA 50, basal diet + 50 mg/kg ursolic acid; UA 200, basal diet + 200 mg/kg ursolic acid; UA 400, basal diet + 400 mg/kg ursolic acid; SEM, standard error of means; VH/CD: villus height/crypt depth.

Values with different superscript letters are statistically different (P < 0.05).

**Table 5.** Effects of ursolic acid at graded concentrations on ileal digestive enzyme activity of broiler chickens (*n *= 8).

Items	Groups				SEM	*P*-values		
	CON	UA 50	UA 200	UA 400		ANOVA	Linear	Quadratic
Amylase U/mg protein	82.94	85.77	84.87	83.18	1.100	0.790	0.988	0.337
Trypsin U/mg protein	114.35b	124.09ab	130.74a	123.27ab	1.736	0.003	0.011	0.004
Lipase U/mg protein	31.08	34.99	33.71	33.95	0.685	0.225	0.229	0.181

Abbreviations: CON, basal diet; UA 50, basal diet + 50 mg/kg ursolic acid; UA 200, basal diet + 200 mg/kg ursolic acid; UA 400, basal diet + 400 mg/kg ursolic acid; SEM, standard error of means.

Values with different superscript letters are statistically different (*P *< 0.05).

### Meat quality

Supplementary Table S5 describes the effects of different UA levels on breast muscle quality. Supplementation with 200 and 400 mg/kg UA resulted in reductions of 11.92% and 12.95%, respectively, in the b_45min_ value of breast muscle (*P *< 0.05), with a significant linear effect as the dosage increased (*P *< 0.05). In contrast, UA supplementation had no significant effect on the quality of leg muscle (*P *> 0.05, Supplementary Table S6).

### Antioxidant indexes

**Figure 4.**
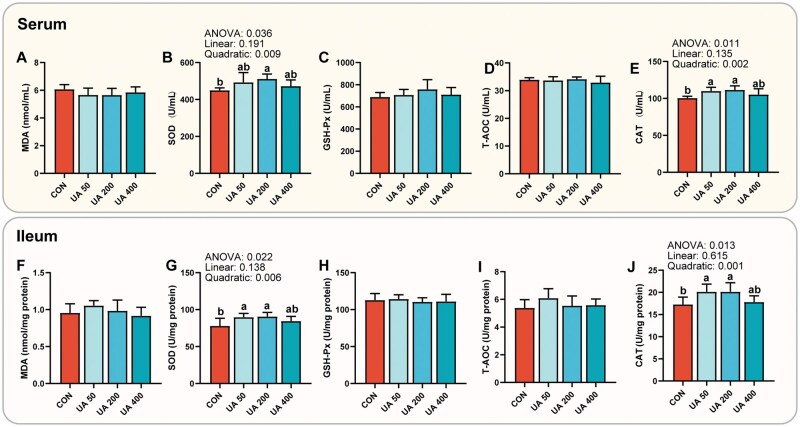
Effects of ursolic acid at graded concentrations on antioxidant capacity of broiler chickens. *n *= 8. Values with different superscript letters are statistically different (ANOVA < 0.05). CON, basal diet; UA 50, basal diet + 50 mg/kg ursolic acid; UA 200, basal diet + 200 mg/kg ursolic acid; UA 400, basal diet + 400 mg/kg ursolic acid; MDA: malondialdehyde; SOD: superoxide dismutase; GSH-Px: glutathione peroxidase; T-AOC: total antioxidant capacity; CAT: catalase.

Figure 4 shows the effects of different UA levels on the antioxidant capacity of broilers. In serum, SOD activity in the UA 200 group was 13.60% higher than that in the CON group (*P *< 0.05), while CAT levels in the UA 50 and UA 200 groups were 9.38% and 10.94% higher than those in the control group, respectively (*P *< 0.05). SOD and CAT exhibited significant quadratic effects with increasing UA supplementation (*P *< 0.05). In the ileum, broilers supplemented with 50 and 200 mg/kg UA showed increases in SOD activity of 15.19% and 16.32%, respectively, and in CAT activity of 16.61% and 16.49%, compared to the control group (*P *< 0.05), with significant quadratic responses to increasing dosage (*P *< 0.05). Due to the significant positive effects of the UA 200 group (hereinafter referred to as the UA group) on intestinal morphology, digestive enzyme activity, and antioxidant capacity, this group was selected for subsequent mechanistic investigations.

### Expression of nutrient transporter genes in the ileum

**Figure 5.**
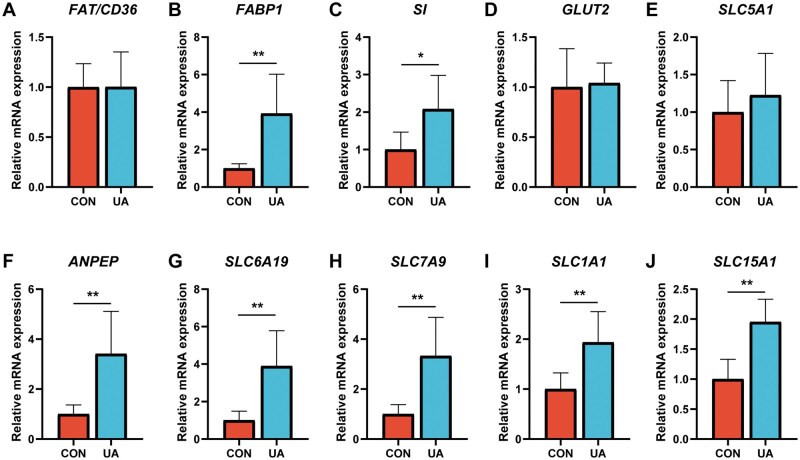
Effect of dietary ursolic acid on the expression genes related to nutrient transporters in ileum. Relative mRNA abundances of (A) fatty acid translocase (*FAT/CD36*), (B) fatty acid binding protein 1 (*FABP1*), (C) sucrase-isomaltase (*SI*), (D) glucose transporters (*GLUT2*), (E) sodium glucose cotransporter-1 (*SLC5A1*), (F) aminopeptidase N (*ANPEP*), (G) neutral amino acid transporter (*SLC6A19*), (H) b0,+amino acid transporter (*SLC7A9*), (I) excitatory amino acid transporter-3 (*SLC1A1*), and (J) peptide transporter protein 1 (*SLC15A1*) in ileum. *n *= 8. * *P *< 0.05, ** *P *< 0.01. CON, basal diet; UA, basal diet + 200 mg/kg ursolic acid.

Figure 5 demonstrates the influence of UA on the expression of nutrient transporter genes in the ileum. Relative to the CON group, the relative expressions of fatty acid binding protein 1 (*FABP1*), sucrase-isomaltase (*SI*), aminopeptidase N (*ANPEP*), neutral amino acid transporter (*SLC6A19*), b0, +amino acid transporter (*SLC7A9*), excitatory amino acid transporter-3 (*SLC1A1*), and peptide transporter protein 1 (*SLC15A1*) genes in the ileum of broilers in the UA group were notably upregulated (*P *< 0.05).

### Expression of antioxidant-related genes in the ileum

**Figure 6.**
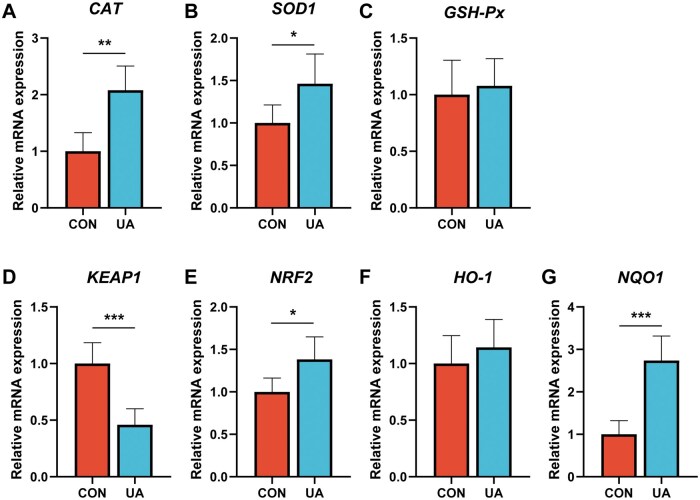
Effect of dietary ursolic acid on the expression genes related to antioxidant in ileum. Relative mRNA abundances of (A) catalase (*CAT*), (B) superoxide dismutase 1 (*SOD1*), (C) glutathione peroxidase (*GSH-Px*), (D) kelch-like ECH-associated protein 1 (*KEAP1*), (E) nuclear factor E2-related factor 2 (*NRF2*), (F) heme oxygenase-1 (*HO-1*), and (G) NAD (P) H: quinone oxidoreductase (*NQO1*) in ileum. *n *= 8. * *P *< 0.05, ** *P *< 0.01, *** *P *< 0.001. CON, basal diet; UA, basal diet + 200 mg/kg ursolic acid.

Figure 6 shows the effects of UA on the expression of antioxidant-associated genes in the ileum. Relative to the CON group, broilers supplemented with UA demonstrated notably upregulated expression levels of catalase (*CAT*), superoxide dismutase 1 (*SOD1*), nuclear factor E2-related factor 2 (*NRF2*), and NAD(P)H: quinone oxidoreductase (*NQO1*) genes in the ileum. Conversely, the expression level of the kelch-like ECH-associated protein 1 (*KEAP1*) gene was significantly reduced (*P *< 0.05).

## Discussion

In recent years, UA, a plant-derived compound with multiple biological functions, has garnered increasing attention for its role in animal nutrition and physiological regulation (Zhang et al., 2023, 2024). The present study demonstrates that dietary supplementation with UA significantly improves growth performance, slaughter traits, lipid metabolism, and antioxidant capacity in broilers. The underlying mechanisms involve enhancements in intestinal function, nutrient transport, and modulation of the KEAP1–NRF2 signaling pathway.

Regarding growth performance, previous research has shown that dietary inclusion of UA increased ADG, improved FCR, and promoted growth in broilers (Zhang et al., 2024). Furthermore, one study reported that supplementation with 500 mg/kg UA significantly enhanced ABW, weight gain rate, and specific growth rate in largemouth bass (*Micropterus salmoides*), while reducing FCR (Wang et al., 2024). Consistent with these findings, the current study observed that the addition of 200 mg/kg UA significantly increased ABW and reduced FCR in broilers. In addition, dietary supplementation with 200 mg/kg UA increased the half-eviscerated yield (%), eviscerated yield (%), and breast muscle yield (%), and significantly elevated serum total protein content. These improvements may be attributed to the ability of UA to enhance protein absorption and stimulate muscle protein synthesis, thereby facilitating muscle development (Deane et al., 2017). The VH/CD ratio is a key indicator of intestinal nutrient absorption capacity (Chen et al., 2024). In this study, broilers in the UA 200 group exhibited a significant increase in the ileal VH/CD ratio and trypsin activity, indicating enhanced digestive and absorptive function (Yang et al., 2018). These results suggest that UA may improve broiler growth performance by enhancing intestinal morphology, increasing protease activity, and promoting protein metabolism.

In terms of lipid metabolism, the present study showed that dietary supplementation with 200 mg/kg UA significantly reduced serum TG and LDL concentrations in broilers. This finding is partially consistent with previous reports (Zhang et al., 2024), further supporting the role of UA in modulating lipid metabolism in broilers. Untargeted metabolomics analysis revealed that UA supplementation significantly altered the serum metabolic profile, particularly affecting lipid metabolites involved in α-linolenic acid metabolism and glycerophospholipid pathways. Specifically, PE levels were significantly increased, while PC levels decreased in the UA group. PE serves as an essential lipid chaperone involved in membrane protein folding and maintenance of membrane integrity (Patel and Witt, 2017), whereas PC is the most abundant phospholipid in serum and plays a central role in lipid absorption, transport, and lipoprotein assembly (Wu et al., 2021). These changes further confirm UA's regulatory effect on lipid metabolism. Moreover, α-linolenic acid—a polyunsaturated fatty acid with antioxidant and lipid-modulating properties (Shahidi and Ambigaipalan, 2018; Elkin and Harvatine, 2023)—was significantly more abundant in the UA-supplemented group. These results indicate that UA may synergistically regulate lipid metabolism and antioxidant status by modulating structural phospholipid metabolism and promoting α-linolenic acid utilization, thereby improving overall lipid homeostasis in broilers.

The regulation of intestinal absorption and transport function represents another key mechanism through which UA improves nutrient metabolism. The efficiency of nutrient absorption is largely governed by specific transporter proteins in intestinal epithelial cells (Yi et al., 2024). In lipid metabolism, *FABP1* is a critical mediator of cellular fatty acid uptake and intracellular transport (Li et al., 2022; Yi et al., 2024). In this study, UA supplementation significantly up-regulated the mRNA expression of *FABP1* in the small intestine, suggesting enhanced fatty acid absorption—a finding consistent with the elevated serum α-linolenic acid levels.

Regarding carbohydrate digestion, SI is responsible for hydrolyzing maltose and sucrose into glucose, which is subsequently absorbed via sodium glucose cotransporter-1 (*SLC5A1*) and glucose transporters (*GLUT*) (England et al., 2024). UA supplementation significantly increased SI mRNA expression but did not affect the transcript levels of *SLC5A1* or *GLUT*, indicating that UA may promote the breakdown of disaccharides without directly influencing glucose uptake.

In protein digestion and absorption, *ANPEP* catalyzes the hydrolysis of N-terminal amino acids from peptides, while *SLC6A19*, *SLC7A9*, *SLC1A1*, and *SLC15A1* mediate the transmembrane transport of free amino acids and small peptides (Teng et al., 2023; Uyanga et al., 2023; Toschi et al., 2024). UA supplementation significantly up-regulated the expression of *ANPEP* and these amino acid and peptide transporter genes. Combined with the previously observed increases in ileal trypsin activity and serum TP content, these results indicate that UA enhances protein hydrolysis and amino acid absorption. Overall, these findings suggest that UA improves nutrient utilization efficiency by enhancing the digestion and absorption of lipids, amino acids, and small peptides, which may represent a key molecular basis for its growth-promoting effects.

Oxidative stress arises from an imbalance between the production of reactive oxygen species and the capacity of the antioxidant defense system, contributing to the development of various diseases (Kim et al., 2024). Broilers are particularly susceptible to oxidative damage due to their rapid growth and exposure to stress factors such as high-density rearing. GSH-Px, CAT, and SOD are major enzymatic antioxidants in broilers and play essential roles in maintaining redox balance (Halliwell, 2024). Multiple studies have confirmed the potent antioxidant properties of UA in poultry (Liu et al., 2022b; Yao et al., 2023; Zhang et al., 2024). In this study, supplementation with 200 mg/kg UA significantly increased serum SOD activity, while both 50 and 200 mg/kg UA markedly elevated SOD and CAT concentrations in the serum and ileum. These results are partially consistent with previous research in which 450 mg/kg UA was shown to enhance SOD and GSH-Px activities in the serum and ileum while reducing MDA levels (Zhang et al., 2024). Variations in certain outcomes may be due to differences in experimental animals, dosage levels, absorption efficiency, and dietary composition.

The KEAP1–NRF2 signaling pathway is a key regulator of redox reactions and an essential component of the endogenous antioxidant defense system (Liu et al., 2022a). NRF2 plays a critical role in maintaining the cellular antioxidant response by driving the expression of downstream antioxidant enzymes. KEAP1 regulates NRF2 by targeting it for proteasomal degradation, thereby suppressing NRF2 signaling (Adinolfi et al., 2023). At the molecular level, this study revealed that UA activates the endogenous antioxidant defense system by downregulating *KEAP1* expression, relieving its inhibition of *NRF2*, and subsequently promoting the expression of downstream antioxidant genes, including *NQO1*, *CAT*, and *SOD1*. These results demonstrate that UA enhances antioxidant capacity in broilers through the KEAP1–NRF2 pathway.

While this study offers valuable insights into the benefits of UA for broilers, certain limitations should be noted. Although untargeted metabolomics indicated UA-modulated pathways, targeted metabolomics is needed to quantify key metabolite changes. In addition, despite qRT-PCR revealing changes in gene expression related to nutrient transport and antioxidant activity, these results require validation at the protein level—for instance, via Western blotting—to confirm translational correspondence. Further studies using these approaches would help solidify the mechanistic insights presented.

## Conclusion

In summary, dietary UA supplementation enhances broiler growth and slaughter performance. These improvements are likely mediated through enhanced ileal morphology, protease activity, and upregulation of fatty acid and amino acid-related transporter genes. UA also modulates serum lipid metabolism via α-linolenic and glycerophospholipid pathways, and boosts antioxidant capacity by activating the KEAP1–NRF2 signaling pathway.

## Supplementary Material

skaf333_Supplementary_Data

## Data Availability

The data that support the study findings are available upon request.

## Abbreviations:

a*: redness
AAs: amino acids
ABW: Average body weight
ADFI: average daily feed intake
ADG: average daily gain
ALB: albumin
ANPEP: aminopeptidase N
b*: yellowness
SLC7A9: b^0^,^+^amino acid transporter
SLC6A19: neutral amino acid transporter
C: complement
CAT: catalase
CATHB: cathelicidins
CD: crypt depth
CON: control
SLC1A1: excitatory amino acid transporter-3
ELISA: enzyme-linked immunosorbent assay
FABP1: fatty acid binding protein 1
FAT/CD36: Fatty acid translocase
FCR: feed conversion ratio
GLB: globulin
GLU: glucose
GLUT: glucose transporters
GSH-Px: glutathione peroxidase
H&E: hematoxylin-eosin
HDL: high-density lipoprotein
HMDB: Human Metabolome Database
KEAP1: kelch-like ECH-associated protein 1
KEGG: Kyoto Encyclopedia of Genes and Genomes
L*: brightness
LDL: low-density lipoprotein
MDA: malondialdehyde
NQO1: NAD(P)H: quinone oxidoreductase
NRF2: nuclear factor E2-related factor 2
OPLS-DA: Orthogonal partial least squares discriminant analysis
PC: phosphatidylcholine
PE: phosphatidylethanolamine
SLC15A1: peptide transporter protein 1
PS: phosphatidylserine
qRT-PCR: Quantitative Real-Time PCR
SLC5A1: sodium glucose cotransporter-1
SI: sucrase-isomaltase
SOD: superoxide dismutase
SPSS: Statistical Package for the Social Sciences
T-AOC: total antioxidant capacity
T-CHO: total cholesterol
TG: triglyceride
TP: total protein
UA: ursolic acid
VH: villus height
VIP: variable importance projection
