# Supplementary material for: Ursolic acid improves growth performance, intestinal health, and antioxidant status in broilers by regulating lipid metabolism and the KEAP1-NRF2 pathway
Source: J Anim Sci. 2025 Sep 29;103:skaf333. doi: 10.1093/jas/skaf333 (PMC12568757; doi:10.1093/jas/skaf333)
Supplement: skaf333_Supplementary_Data [file skaf333_supplementary_data.zip › Revised Supplementary file.docx]

**Supplementary Material S1. Slaughter performance.**

The abdominal cavity was quickly opened, and organs were separated and feathers, keratin, sharp beaks, and blood were removed. All carcasses were weighed to calculate the dressing percentage, half-eviscerated yield and eviscerated yield, breast muscle yield, leg muscle yield and abdominal fat yield. Dressing percentage was determined by multiplying the ratio of dressed weight to body weight (BW) by 100.

All yields were calculated as follows:

1. Dressed weight = BW – (blood + feather) weights.

2. Dressing percentage (%) = 100 × dressed weigh/BW.

3. Half-eviscerated weight = dressed weight – (trachea + esophagus + crop + intestine + spleen + pancreas + gallbladder + reproductive organ + contents and membrane of gizzard) weights; Half-eviscerated yield (%) = 100 × half-eviscerated weight/BW.

4. Eviscerated weight = half-eviscerated weight – (heart + liver + proventriculus + gizzard + fat around abdomen and gizzard + head + neck + claw) weights; Eviscerated yield (%) = 100 × eviscerated weight/BW.

5. Breast muscle yield (%) = 100 × breast muscle weight/eviscerated weight.

6. Leg muscle yield (%) = 100 × thigh muscle weight/eviscerated weight.

7. Abdominal fat yield (%) = 100 × fat around abdomen and gizzard/eviscerated weight.

**Supplementary Material S2. Intestinal Morphology.**

The collected segment was flushed with phosphate-buffered saline (PBS) and fixed in 4% paraformaldehyde. Ileum tissue specimens were embedded in paraffin, and transverse sections of 4 μm thickness were generated for hematoxylin-eosin (H&E) staining. Section images were acquired using a BA210Digital trinocular digital microscope (Macoptics Industrial Group Co., Ltd.). Villus height (VH), crypt depth (CD), villus width, and intestinal wall thickness were quantitatively analyzed in five well-oriented villi per section using ImageJ software. The villus height-to-crypt depth ratio (VH/CD) was calculated, and the average of the five villi was used as the final measurement data.

**Supplementary Material S3.** **Digestive Enzyme Assays.**

At the end of the 42-day trial, ileal digesta were promptly collected using a sterile surgical blade. The weight of the digesta was recorded, and samples were homogenized in ice-cold phosphate-buffered saline (PBS) at a ratio of 1:9 (w/v). The homogenate was subsequently centrifuged at 4 °C for 15 minutes. Finally, the enzyme activity in the supernatant was determined according to the manufacturer's instructions of the specific assay kit.

**Supplementary Material S4. Meat Quality.**

Drip Loss: To determine drip loss (%), triplicate samples of pectoral and leg muscle were individually placed in sealed plastic bags and stored at 4 °C for 24 hours. The difference between the initial and final weight of each sample was calculated and expressed as a percentage of the initial weight.

Cooking Loss: Fresh muscle slices measuring 4 × 3 × 1 cm³ were weighed and sealed in plastic bags. These samples were cooked in an 80 °C water bath until an internal temperature of 70 °C was reached (approximately 15 minutes). After cooking, the slices were cooled in water, blotted dry, and reweighed.

Cooking loss was calculated as follows:

(initial weight−final weight)/initial weight×100%

Shear Force: Meat samples were first cooked in a constant-temperature water bath at 80 °C. After cooking, the meat was cut into cubes measuring 2.5 cm (length) × 1.0 cm (width) × 0.25 cm (height). Shear force was measured at three independent locations on each sample using an RH-N50 Meat Tenderness Tester (Runhu Instrument Co., Ltd., Guangzhou, China). The values obtained from the three measurements were averaged to determine the final shear force.

**Supplementary Material S5. Quantitative Real-Time PCR (qRT-PCR) Analysis**

Total RNA was isolated using TRIzol reagent (Vazyme Biotech, Nanjing, China). RNA concentration and purity were assessed with the NanoDrop 2000 (Thermo Fisher Scientific, Waltham, MA, USA), while integrity was confirmed by electrophoresis on a 1% agarose gel. For cDNA synthesis, total RNA was reverse-transcribed using the PrimeScripTM RT Reagent Kit (Takara Bio, Shiga, Japan). Subsequent qRT-PCR was performed using TB GreenTM Premix Ex TaqTM II (Takara Bio, Shiga, Japan) on a Bio-Rad CFX96 Real-Time PCR system (Hercules, CA, USA). The relative mRNA expression was quantified using the 2^−ΔΔCT^ method.

**Table S1.** Composition and nutrient levels of the basal diet (air-dry basis) %

| **Items** | **Trial period** | |
| --- | --- | --- |
|  | **1~21 d** | **22~42 d** |
| Ingredients |  |  |
| Corn | 48.47 | 50.19 |
| Soybean meal | 30.03 | 30.55 |
| Puffed soybeans | 10.00 | 5 |
| Vegetable oil | 3.20 | 6.06 |
| Premix^1^ | 5.00 | 5 |
| Limestone | 1.60 | 1.5 |
| Calcium phosphate | 1.25 | 1.25 |
| Sodium chloride | 0.35 | 0.35 |
| Choline chloride | 0.10 | 0.1 |
| Total | 100.00 | 100.00 |
| Nutrient levels^2^ |  |  |
| Metabolic energy /(MJ/kg) | 12.96 | 13.59 |
| Crude protein | 23.11 | 21.28 |
| Crude fibre | 3.46 | 3.14 |
| Calcium | 0.85 | 0.70 |
| Available phosphorus | 0.42 | 0.37 |
| Total phosphorus | 0.65 | 0.55 |
| Lysine | 1.25 | 1.12 |
| Methionine | 0.52 | 0.49 |
| Threonine | 0.91 | 0.84 |

^1^The premix provided the following per kg of diets: vitamin A, 10,000 IU; vitamin D3, 4,000 IU; vitamin E, 20 IU; vitamin K3, 2 mg; vitamin B_1_, 2 mg; vitamin B_2_, 6 mg; vitamin B_6_, 3 mg; vitamin B_12_, 0.02 mg; nicotinamide, 40 mg; calcium pantothenate, 10 mg; folic acid, 1 mg; biotin, 0.12 mg; Cu, 16 mg; Fe, 80 mg; Zn, 110 mg; Mn, 120 mg; I, 1.5 mg; Se, 0.3 mg.

^2^Crude protein, calcium and total phosphorus in nutrients are measured values, while the rest are calculated values.

**Table S2.** Gene-specific primer sequences used for gene transcription analyses.

| Gene | Genebank accession number | Primers sequences (5’→3’) | Product size (bp) |  |
| --- | --- | --- | --- | --- |
| *ACTB* | NM_205518.1 | F: CAACACAGTGCTGTCTGGTGGTA | 205 |  |
|  |  | R: ATCGTACTCCTGCTTGCTGATCC |  |  |
| *FAT/CD36* | NM_001030731.1 | F: CTGGGAAGGTTACTGCGATT | 178 |  |
|  |  | R: GCGAGGAACTGTGAAACGATA |  |  |
| *FABP1* | NM_204192.4 | F: TGGGGAAGAGTGTGAGATG | 144 |  |
|  |  | R: ATTGTATGGGTGATGGTGTCT |  |  |
| *SI* | XM_015291762.1 | F: CGCAAAAGCACAGGGACAGT | 138 |  |
|  |  | R: TCGATACGTGGTGTGCTCAGTT |  |  |
| *GLUT2* | NM_207178.1 | F: AAAGCAAAATGCAGGCGGAG | 132 |  |
|  |  | R: GTGCTTCTATCACCTTCTGCG |  |  |
| *SLC5A1* | NM_001293240.2 | F: CCACCGCCATAAGGATCAACA | 101 |  |
|  |  | R: GTTGGTTGAGTACATAGCCCATAC |  |  |
| *ANPEP* | | NM_001013611.2 | F: AATACGCGCTCGAGAAAACC | 70 |
|  |  |  | R: AGCGGGTACGCCGTGTT |  |
| *SLC6A19* | XM_419056.5 | F: GTGTTTGGAACCCTAAATACGAGG | 72 |  |
|  |  | R: TAGCATAGACCCAGCCAGGA |  |  |
| *SLC7A9* | NM_001199133.1 | F: CAGTAGTGAATTCTCTGAGTGTGAAGCT | 88 |  |
|  |  | R: GCAATGATTGCCACAACTACCA |  |  |
| *SLC1A1* | XM_424930.5 | F: TGCTGCTTTGGATTCCAGTGT | 79 |  |
|  |  | R: AGCAATGACTGTAGTGCAGAAGTAATATATG |  |  |
| *SLC15A1* | AY029615.1 | F: TACGCATACTGTCACCATCA | 205 |  |
|  |  | R: TCCTGAGAACGGACTGTAAT |  |  |
| *CAT* | NM_001031215.2 | F: TCAGGAGATGTGCAGCGTTT | 109 |  |
|  |  | R: TCTTACACAGCCTTTGGCGT |  |  |
| *SOD1* | NM_205064.1 | F: GGCAATGTGACTGCAAAGGG | 133 |  |
|  |  | R: CCCCTCTACCCAGGTCATCA |  |  |
| *GSH-Px* | | NM_001277853.1 | F: GCTGTTCGCCTTCCTGAGAG | 118 |
|  |  |  | R: GTTCCAGGAGACGTCGTTGC |  |
| *KEAP1* | XM_025145847.1 | F: GGTTACGATGGGACGGATCA | 135 |  |
|  |  | R: CACGTAGATCTTGCCCTGGT |  |  |
| *NRF2* | NM_001007858.1 | F: GAGCCCATGGCCTTTCCTAT | 210 |  |
|  |  | R: CACAGAGGCCCTGACTCAAA |  |  |
| *HO-1* | NM_205344.1 | F: AAACTTCGCAGCCACACAAC | 155 |  |
|  |  | R: GACCAGCTTGAACTCGTGGA |  |  |
| *NQO1* | NM_001277619.2 | F: CCATCTCTGACCTCTACGCC | 178 |  |
|  |  | R: AGGTCAGCCGCTTCAATCTT |  |  |

**Table S3.** Effects of ursolic acid at graded concentrations on mortality rate of broiler chickens (*n* = 80).

| Items | Groups | | | | χ^2^ | *P*-value |
| --- | --- | --- | --- | --- | --- | --- |
|  | CON | UA 50 | UA 200 | UA 400 |  |  |
| Mortality rate (%) | 5.00 | 2.50 | 3.75 | 3.75 | 1.632 | 0.452 |

**Table S4.** Effects of ursolic acid at graded concentrations on organ development of broiler chickens (*n* = 8).

| Items | Groups | | | | SEM | *P*-values | | |
| --- | --- | --- | --- | --- | --- | --- | --- | --- |
|  | CON | UA 50 | UA 200 | UA 400 |  | *P* | Linear | Quadratic |
| Weight (g) |  |  |  |  |  |  |  |  |
| Heart | 5.83 | 6.05 | 6.36 | 6.20 | 0.111 | 0.378 | 0.233 | 0.201 |
| Liver | 28.13 | 27.55 | 28.61 | 31.33 | 0.605 | 0.121 | 0.026 | 0.415 |
| Spleen | 0.89 | 0.94 | 1.19 | 1.04 | 0.050 | 0.148 | 0.175 | 0.070 |
| Kidney | 6.93 | 6.79 | 7.08 | 6.80 | 0.353 | 0.992 | 0.971 | 0.833 |
| Bursa of Fabricius | 2.16 | 1.80 | 1.95 | 2.26 | 0.102 | 0.392 | 0.382 | 0.259 |
| Index (%) |  |  |  |  |  |  |  |  |
| Heart | 0.59 | 0.61 | 0.63 | 0.61 | 0.010 | 0.671 | 0.607 | 0.277 |
| Liver | 2.88 | 2.79 | 2.83 | 3.08 | 0.057 | 0.278 | 0.113 | 0.267 |
| Spleen | 0.09 | 0.10 | 0.12 | 0.10 | 0.005 | 0.256 | 0.294 | 0.103 |
| Kidney | 0.71 | 0.69 | 0.70 | 0.67 | 0.034 | 0.984 | 0.745 | 0.932 |
| Bursa of Fabricius | 0.22 | 0.18 | 0.19 | 0.22 | 0.010 | 0.376 | 0.646 | 0.164 |

Abbreviations: CON, basal diet; UA 50, basal diet + 50 mg/kg ursolic acid; UA 200, basal diet + 200 mg/kg ursolic acid; UA 400, basal diet + 400 mg/kg ursolic acid; SEM, standard error of means.

**Table S5.** Effects of ursolic acid at graded concentrations on breast muscle quality of broiler chickens (*n* = 8).

| Items | Groups | | | | SEM | *P*-values | | |
| --- | --- | --- | --- | --- | --- | --- | --- | --- |
|  | CON | UA 50 | UA 200 | UA 400 |  | *P* | Linear | Quadratic |
| Breast muscle |  |  |  |  |  |  |  |  |
| pH_45min_ | 6.46 | 6.56 | 6.66 | 6.59 | 0.056 | 0.663 | 0.460 | 0.321 |
| pH_24h_ | 5.90 | 5.93 | 5.94 | 5.94 | 0.026 | 0.947 | 0.651 | 0.739 |
| pH_48h_ | 5.95 | 5.90 | 5.83 | 5.92 | 0.028 | 0.477 | 0.759 | 0.128 |
| Drip loss_24 h_ (%) | 4.53 | 4.62 | 2.64 | 2.80 | 0.441 | 0.223 | 0.077 | 0.371 |
| Cooking loss (%) | 34.37 | 34.37 | 36.31 | 32.51 | 0.983 | 0.622 | 0.560 | 0.266 |
| Shear force (kg.f) | 3.61 | 3.06 | 3.79 | 4.45 | 0.202 | 0.104 | 0.032 | 0.529 |
| L_45min_ | 46.34 | 41.54 | 43.48 | 40.87 | 0.981 | 0.197 | 0.154 | 0.826 |
| a_45min_ | 5.51 | 6.71 | 5.85 | 6.05 | 0.219 | 0.276 | 0.966 | 0.736 |
| b_45min_ | 5.79^a^ | 5.61^ab^ | 5.10^b^ | 5.04^b^ | 0.102 | 0.012 | 0.003 | 0.166 |
| L_24h_ | 43.42 | 44.69 | 49.14 | 44.94 | 1.517 | 0.592 | 0.656 | 0.208 |
| a_24h_ | 1.62 | 1.81 | 2.04 | 1.84 | 0.190 | 0.907 | 0.713 | 0.528 |
| b_24h_ | 6.72 | 5.47 | 6.27 | 4.61 | 0.297 | 0.053 | 0.035 | 0.172 |
| L_48h_ | 47.00 | 44.71 | 46.91 | 43.55 | 0.673 | 0.190 | 0.174 | 0.392 |
| a_48h_ | 1.34 | 1.75 | 1.51 | 1.82 | 0.171 | 0.767 | 0.513 | 0.940 |
| b_48h_ | 6.55 | 5.75 | 6.33 | 5.17 | 0.244 | 0.180 | 0.105 | 0.501 |

Abbreviations: CON, basal diet; UA 50, basal diet + 50 mg/kg ursolic acid; UA 200, basal diet + 200 mg/kg ursolic acid; UA 400, basal diet + 400 mg/kg ursolic acid; SEM, standard error of means; L: lightness; b: yellowness; a: redness.

Values with different superscript letters are statistically different (*P* < 0.05).

**Table S6.** Effects of ursolic acid at graded concentrations on leg muscle quality of broiler chickens (*n* = 8).

| Items | Groups | | | | SEM | *P*-values | | |
| --- | --- | --- | --- | --- | --- | --- | --- | --- |
|  | CON | UA 50 | UA 200 | UA 400 |  | *P* | Linear | Quadratic |
| Leg muscle |  |  |  |  |  |  |  |  |
| pH_45min_ | 6.30 | 6.38 | 6.31 | 6.26 | 0.038 | 0.747 | 0.453 | 0.665 |
| pH_24h_ | 6.24 | 6.37 | 6.29 | 6.13 | 0.050 | 0.404 | 0.220 | 0.365 |
| pH_48h_ | 6.37 | 6.35 | 6.26 | 6.15 | 0.042 | 0.248 | 0.046 | 0.983 |
| Drip loss_24 h_ (%) | 3.07 | 1.80 | 1.38 | 2.00 | 0.322 | 0.299 | 0.382 | 0.128 |
| Cooking loss (%) | 30.98 | 32.85 | 34.13 | 35.00 | 0.680 | 0.175 | 0.044 | 0.449 |
| Shear force (kg.f) | 3.40 | 2.44 | 3.61 | 3.08 | 0.179 | 0.098 | 0.711 | 0.519 |
| L_45min_ | 46.36 | 45.59 | 48.11 | 46.75 | 0.612 | 0.548 | 0.532 | 0.358 |
| a_45min_ | 7.04 | 7.21 | 6.72 | 7.19 | 0.313 | 0.201 | 0.368 | 0.354 |
| b_45min_ | 7.26 | 7.00 | 5.70 | 7.25 | 0.257 | 0.092 | 0.832 | 0.016 |
| L_24h_ | 46.52 | 45.94 | 49.04 | 48.02 | 0.536 | 0.155 | 0.119 | 0.205 |
| a_24h_ | 6.93 | 6.19 | 4.27 | 5.06 | 0.580 | 0.388 | 0.210 | 0.237 |
| b_24h_ | 6.78 | 5.82 | 5.68 | 6.04 | 0.334 | 0.682 | 0.620 | 0.359 |
| L_48h_ | 47.53 | 45.14 | 46.21 | 46.47 | 0.545 | 0.507 | 0.926 | 0.476 |
| a_48h_ | 7.03 | 5.48 | 4.02 | 6.33 | 0.444 | 0.088 | 0.689 | 0.013 |
| b_48h_ | 7.70 | 5.49 | 5.67 | 6.68 | 0.374 | 0.128 | 0.752 | 0.067 |

Abbreviations: CON, basal diet; UA 50, basal diet + 50 mg/kg ursolic acid; UA 200, basal diet + 200 mg/kg ursolic acid; UA 400, basal diet + 400 mg/kg ursolic acid; SEM, standard error of means; L: lightness; b: yellowness; a: redness.

Values with different superscript letters are statistically different (*P* < 0.05).

**
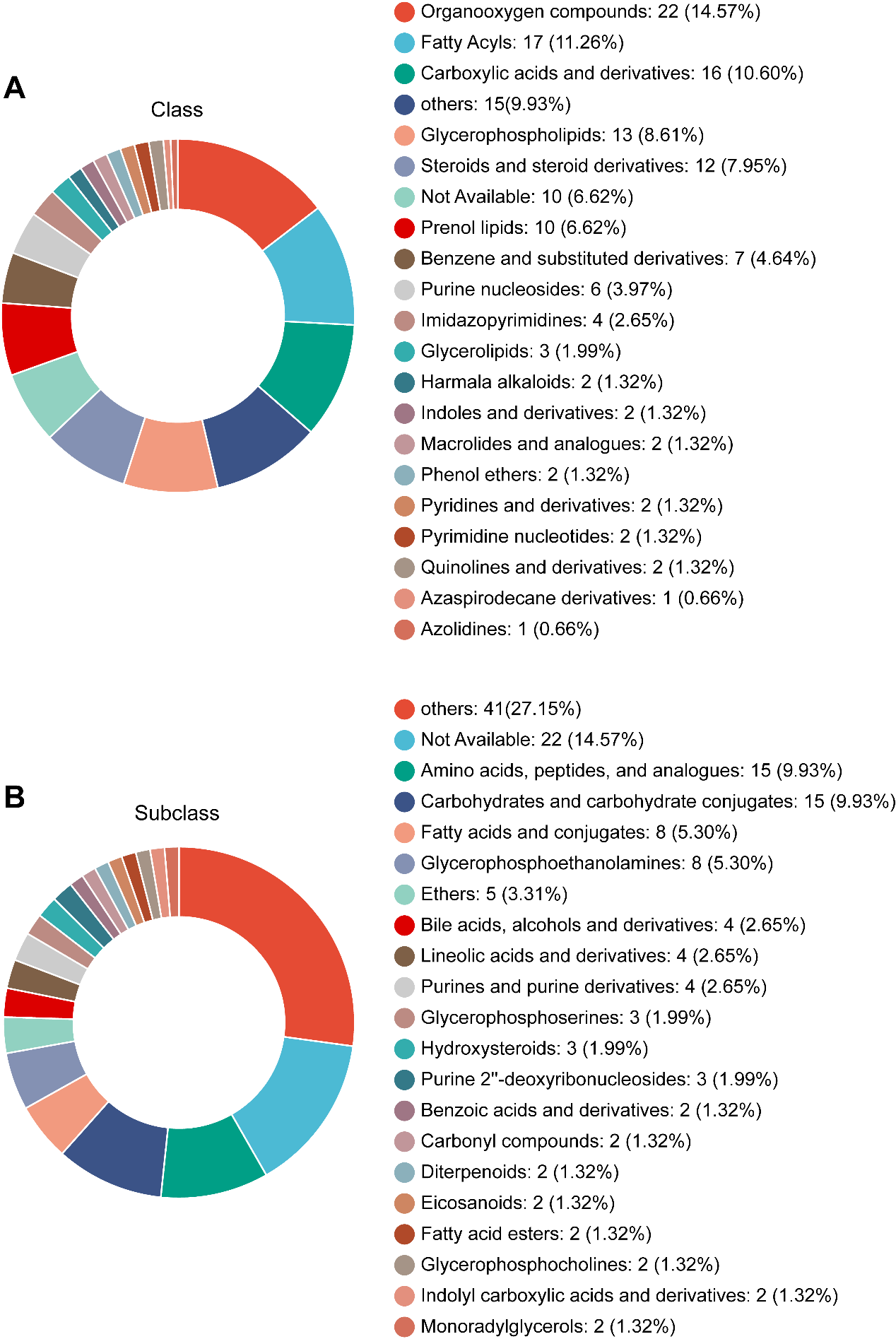
**

**Figure S1.** Human Metabolome Database (HMDB) classification of differential metabolites. (A) Class level. (B) Subclass level. *n* = 8.
